# Predictors of Postoperative Seizure Recurrence: A Longitudinal Study of Temporal and Extratemporal Resections

**DOI:** 10.1155/2016/7982494

**Published:** 2016-03-16

**Authors:** Hai Chen, Pradeep N. Modur, Niravkumar Barot, Paul C. Van Ness, Mark A. Agostini, Kan Ding, Puneet Gupta, Ryan Hays, Bruce Mickey

**Affiliations:** ^1^Department of Neurology and Neurotherapeutics, University of Texas Southwestern Medical Center, Dallas, TX 75390, USA; ^2^Comprehensive Epilepsy Center, NYU Langone Medical Center, New York, NY 10016, USA; ^3^Seton Brain and Spine Institute, The University of Texas at Austin, Austin, TX 78701, USA; ^4^Department of Neurology, Baylor College of Medicine Medical Center, Houston, TX 77030, USA; ^5^Neurology Consultants of Dallas, PA, Dallas, TX 75231, USA; ^6^Department of Neurosurgery, University of Texas Southwestern Medical Center, Dallas, TX 75390, USA

## Abstract

*Objective*. We investigated the longitudinal outcome of resective epilepsy surgery to identify the predictors of seizure recurrence.* Materials and Methods*. We retrospectively analyzed patients who underwent resections for intractable epilepsy over a period of 7 years. Multiple variables were investigated as potential predictors of seizure recurrence. The time to first postoperative seizure was evaluated using survival analysis and univariate analysis at annual intervals.* Results*. Among 70 patients, 54 (77%) had temporal and 16 (23%) had extratemporal resections. At last follow-up (mean 48 months; range 24–87 months), the outcome was Engel class I in 84% (*n* = 59) of patients. Seizure recurrence followed two patterns: recurrence was “early” (within 2 years) in 82% of patients, of whom 83% continued to have seizures despite optimum medical therapy; recurrence was “late” (after 2 years) in 18%, of whom 25% continued to have seizures subsequently. Among the variables of interest, only resection site and ictal EEG remained as independent predictors of seizure recurrence over the long term (*p* < 0.05). Extratemporal resection and discordance between ictal EEG and resection area were associated with 4.2-fold and 5.6-fold higher risk of seizure recurrence, respectively.* Conclusions*. Extratemporal epilepsy and uncertainty in ictal EEG localization are independent predictors of unfavorable outcome. Seizure recurrence within two years of surgery indicates poor long-term outcome.

## 1. Introduction

Epilepsy is a common disorder with an incidence of 50/100,000 per year and a prevalence of 5–10/1000 in North America [1]. About 30% epilepsy patients are intractable to antiepileptic medication treatment [2]. Randomized controlled trials have established resective surgery as an effective treatment for intractable epilepsy [3, 4].

Despite improved outcome, seizure recurrence after resective surgery is not uncommon. A multicenter study demonstrated that only 50–68% of patients remained completely seizure-free after anterior temporal lobectomy (ATL) with two or more years of follow-up [5]. Multiple predictors of postoperative seizure recurrence have been identified, including older age at surgery, extratemporal seizure onset, discordant ictal and interictal findings, previous history of secondary generalized convulsive seizures, normal MRI, widespread imaging abnormalities, early postoperative seizures, and prolonged seizure history [6–10]. However, the published literature on epilepsy surgery outcome is limited by several factors: first, comparison of different studies shows conflicting results for the same variables making it difficult to draw definitive conclusions; second, many studies have employed cross-sectional designs that do not address temporal changes in postoperative outcome, potentially leading to conflicting results and conclusions among studies with different follow-up periods; third, the studies have tended to address outcome in selected subgroups defined by pathology (e.g., mesial temporal sclerosis (MTS)) or resection site (e.g., temporal lobe epilepsy (TLE), frontal lobe epilepsy) rather than the entire group of patients undergoing epilepsy surgery, which is more clinically meaningful in terms of success of epilepsy surgery [7, 8, 10–13]. Recognizing these limitations, we sought to investigate the longitudinal outcome of epilepsy surgery over time, regardless of the presumptive diagnosis or resection site. Our goal was to identify the predictors of seizure recurrence in patients who underwent temporal and extratemporal resective epilepsy surgery.

## 2. Materials and Methods 

### 2.1. Patient Selection and Presurgical Evaluation

From the epilepsy database, we retrospectively identified the patients who underwent resective surgery between January 2006 and July 2012 at Parkland Memorial Hospital, affiliated with the comprehensive epilepsy program at the University of Texas Southwestern Medical Center. All patients had inpatient video-EEG monitoring and brain MRI. Positron emission tomography (PET), single-photon emission computed tomography (SPECT), neuropsychological assessment, and Wada test were performed in selected patients depending on clinical indication. Invasive monitoring with subdural grid electrodes or depth electrodes was performed when the scalp EEG findings were inconclusive. Intraoperative electrocorticography (ECoG) was done in selected patients to further tailor the resection area. The patients were discussed in the multidisciplinary epilepsy conference to reach consensus before proceeding with the surgery. The resected tissue was evaluated by experienced neuropathologists. We included patients who underwent surgery for intractable epilepsy during the above period and had at least 2 years of postoperative follow-up in our center. We excluded patients whose postoperative pathology confirmed high-grade malignant tumor because their outcome could be significantly influenced by the underlying tumor itself.

From chart review, we extracted the following demographic and clinical data: age, gender, preoperative seizure frequency, epilepsy duration prior to surgery, history of secondary generalized tonic-clonic seizures (SGTCS), number of antiepileptic drugs (AEDs) tried prior to surgery, resection site, ictal EEG findings, interictal EEG findings, imaging findings, Wada memory lateralization, and pathological findings. We did not consider auras in estimating the seizure frequency. MRI was considered as abnormal only if the observed findings were consistent with well-established, potentially epileptogenic entities; in other words, isolated abnormalities that are unlikely to cause seizures (e.g., chronic microvascular disease and nonspecific white matter changes) were not considered as abnormal. We classified the resection site as temporal or extratemporal. We determined the ictal onsets based on established criteria [14–16]. With respect to the resection site, we classified the ictal and interictal EEG findings as concordant (i.e., strictly confined to the resection area) or discordant (i.e., any evidence of a wider, even if lateralized, spatial distribution outside the resection area including more than one seizure onset zone). The Wada memory was classified as concordant with the resection site if the surgical hemisphere showed poorer memory function compared with the nonsurgical side as noted by ≥20% difference in the total recall of items; all other Wada results were classified as discordant.

### 2.2. Outcome Assessment

We reviewed the charts of patients who had at least 2 years of postoperative follow-up. Seizure outcome was assessed using Engel classification [17]. Outcome was classified as class I (seizure-free or free of disabling seizures); class II (rare disabling seizures); class III (worthwhile improvement); and class IV (no worthwhile improvement). Specific to this study, the outcome was further stratified as seizure-free (class I) or seizure recurrence (classes II–IV). Presence of only isolated auras postoperatively was not considered to indicate seizure recurrence.

Longitudinal outcome was evaluated at annual intervals. Outcome at the 2-year interval was classified as class I if the patients remained seizure-free for the 2-year period prior to the follow-up visit. Starting at the 3rd postoperative year, the outcome was classified as class I if the patients remained seizure-free for the 1-year period prior to the follow-up visit. The time to first postoperative seizure was evaluated (see below). Immediate postoperative seizures, within 1 month after surgery, were not included in the analysis.

### 2.3. Statistical Analysis

The data range and median values were summarized for the continuous variables such as age, number of AEDs, preoperative seizure frequency, and duration of epilepsy. Continuous variables were converted into categorical variables by grouping the values into categories for univariate analysis using chi-square or Fisher's exact tests as appropriate. Variables with *p* values < 0.05 on univariate analysis were then tested in a multivariate Cox regression test to obtain hazard ratios and 95% confidence intervals (CI). Kaplan-Meier survival analysis was used to evaluate longitudinal seizure outcome. Statistical significance of survival analysis was tested by log-rank tests. All statistical analyses were performed using SPSS 10.0 (IBM Corp., Armonk, NY, USA).

## 3. Results

### 3.1. Patient Characteristics

There were 78 patients eligible for inclusion in the study. Of these, 8 patients were excluded because the postoperative pathology was consistent with high-grade malignant tumor. Thus, 70 patients (32 males and 38 females) were available for analysis (Table 1). The age at epilepsy surgery ranged from 21 to 64 years (mean 39 years). Preoperative epilepsy duration ranged from 1 to 57 years (mean 18 years). Forty-six patients (66%) had history of SGTC seizures. Patients had tried multiple AEDs prior to surgery (range 1–10; mean 5.1). Fifty-four patients (77%) had temporal resections (including ATL and lesionectomy in the temporal lobe), whereas 16 (23%) had extratemporal resections (frontal, *n* = 14, and parietal, *n* = 2). Twelve patients had extraoperative invasive grid or depth electrode evaluation before resection. Forty-one patients had intraoperative ECoG. Ictal EEG findings were concordant in 52 (74%), discordant in 16 (23%), and inconclusive (not recorded) in 2 (3%) patients. Interictal findings were concordant in 37 (53%), discordant in 25 (36%), and normal in 8 (11%) patients. MRI was normal in 17 (24%) and abnormal in 53 (76%) patients; the abnormalities included MTS (*n* = 34), mass lesion (*n* = 8), cavernoma (*n* = 3), encephalomalacia (*n* = 3), and other (*n* = 5). Wada test was performed in 52 patients; in 44 (85%) patients, the memory lateralization was concordant with the surgical side. Pathology showed mesial temporal sclerosis (MTS) or gliosis (*n* = 45, 64%), benign tumor (*n* = 7, 10%), vascular lesion (*n* = 4, 6%), focal cortical dysplasia (*n* = 1, 1%), other abnormality (*n* = 6, 9%), and no abnormality (*n* = 7, 10%).

### 3.2. Temporal Patterns of Postoperative Seizure Recurrence

The follow-up period for assessment of seizure recurrence ranged from 24 to 87 months (mean 48.1; median 43.5 months). At the last follow-up visit, the outcome was class I in 59 (84%) patients (temporal, *n* = 49; extratemporal, *n* = 10) and classes II–IV in 11 (16%) patients (temporal, *n* = 5; extratemporal, *n* = 6).

During the follow-up period, 22 patients experienced seizure recurrence. Using a 2-year cut-off period, we found that the seizure recurrence followed two patterns that were clearly different (*p* < 0.05). In other words, 18/22 patients (82%) had recurrence within 2 years of surgery (“early” recurrence), whereas 4/22 patients (18%) had recurrence after 2 years (“late” recurrence). Among those with early recurrence, 15 (83%) patients continued to have seizures, whereas 3 patients became seizure-free with medication management during subsequent follow-up (Table 2). On the contrary, among those patients with late recurrence, only one (25%) patient continued to have seizures, whereas the other 3 patients became seizure-free with medication management (Table 2). All the patients who regained seizure freedom were reclassified as class I at their subsequent follow-up visits. These findings suggest that early seizure recurrence, within 2 years of surgery, predicts poor long-term outcome.

### 3.3. Univariate Analysis of Predictors of Seizure Recurrence

We analyzed the postoperative outcome (seizure-free versus seizure recurrence) at various follow-up periods using univariate analysis (Table 3). Among the variables of interest, the nonpredictors of seizure recurrence were age, gender, history of SGTC seizures, epilepsy duration, preoperative seizure frequency, number of AEDs, MRI findings, interictal EEG findings, Wada memory lateralization, and lesion pathology. Extratemporal resection predicted seizure recurrence at 2-, 3-, and 4-year intervals (*p* < 0.05). Discordance between ictal EEG localization and resection site predicted seizure recurrence at 2-, 3-, 4-, and 5-year intervals (*p* < 0.05). Outcome beyond 5 years could not be determined due to small sample size. Of note, among the 7 patients with normal pathology, 6 were seizure-free at last follow-up visit, whereas 1 had never achieved seizure freedom.

### 3.4. Multivariate Analysis of Predictors of Seizure Recurrence

Upon multivariate analysis using Cox proportional analysis, only two variables, resection site and ictal EEG, still retained their significance as independent predictors of seizure recurrence (Table 4). Extratemporal resection was associated with a 4.2-fold higher risk of seizure recurrence compared with temporal resection (95% CI 1.5–11). Discordance between ictal EEG and resection area was associated with a 5.6-fold higher risk of seizure recurrence compared with concordance between the two (95% CI 2.0–15.7).

### 3.5. Survival Analysis of Long-Term Seizure Outcome

**Figure 1:**
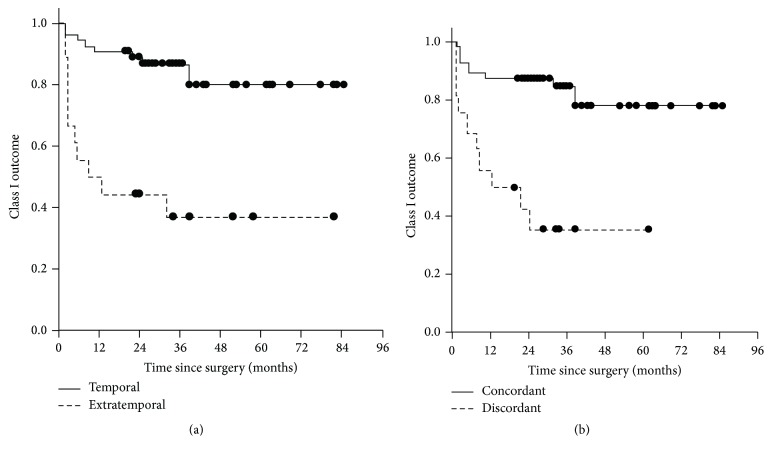
Kaplan-Meier survival analysis of class I seizure outcome: (a) shows the comparison between temporal resection (solid line) and extratemporal resection (dashed line); (b) shows the comparison between concordant ictal EEG (solid line) and discordant ictal EEG (dashed line) with respect to the resection site.

Kaplan-Meier survival analysis demonstrated statistically significant differences in seizure outcome with regard to resection site and ictal EEG findings (Figure 1). Temporal resections (versus extratemporal resections) and concordance between ictal EEG and resection site (versus discordance) were associated with class I seizure outcome over the long-term follow-up intervals (*p* < 0.05).

## 4. Discussion

In this study, we present our single-center experience of longitudinal seizure outcome after epilepsy surgery in a heterogeneous group of 70 patients regardless of resection site or presumptive etiology. The main findings were as follows: (1) >80% of the patients experienced class I outcome at the last mean follow-up of 4 years; (2) in patients with seizure recurrence, the majority of recurrences (>80%) occurred early (within 2 years after surgery) and a majority of such patients (>80%) continued to have seizures over the subsequent follow-up period despite medical management; and (3) among multiple variables, extratemporal resection (versus temporal resection) and discordance between ictal EEG and resection area (versus concordance between the two) predicted 4.2-fold and 5.6-fold higher risk of seizure recurrence over time, respectively.

### 4.1. Seizure Outcome and Temporal Patterns of Seizure Recurrence

In our group of patients who had both temporal and extratemporal resections, class I outcome was achieved in 84% at the last follow-up period (mean 48 months). These results are similar to the previous studies and meta-analysis [18] and indicate that epilepsy surgery, regardless of resection site, is beneficial in patients with medically intractable epilepsy.

Analysis of seizure recurrence patterns in our study showed that the majority of seizure recurrence (82%) occurred within 2 years after surgery, which we chose as the cut-off for “early” recurrence. This early recurrence predicted poor long-term outcome in our study, with a majority (83%) of such patients continuing to have seizures despite optimum medical management. Previous studies of temporal lobectomy demonstrated an initial phase of steep seizure recurrence at about 1-2 years, followed by a relapse rate of 2–5% per year for 5 years before stable seizure freedom was achieved [19, 20]. Using a 6- to 12-month cut-off, other authors have hypothesized that the early seizure recurrences were due to errors in localization of the epileptic focus or incomplete resection, whereas late recurrences were due to* de novo* epileptogenesis [21, 22]. Although our cut-off of 2 years is longer, it provides a practical timeline considering the widely accepted practice of tapering AEDs in patients who have achieved 2 years of postoperative seizure freedom. Our results suggest that one should exercise caution when attempting to proceed with AED simplification if there has been seizure recurrence within 2 years of surgery.

### 4.2. Predictors and Nonpredictors of Seizure Recurrence

Studies of predictors of postoperative seizure recurrence are helpful in selecting the best surgical candidates. Extensive research regarding the predictors of postoperative outcome has been done, and multiple positive or negative predictors have been proposed [23]. However, the literature shows conflicting results, often related to methodological issues, preventing direct clinical application. For example, cross-sectional studies used the last follow-up (which was variable within the group) or the follow-up at a specific postoperative anniversary (e.g., 2 years) as cut-off points to assess outcome [24]. Such studies fail to address the changes in outcome over time due to running down phenomenon, seizure recurrence, and transient improvement or fluctuation [8]. Similarly, studies that have focused on specific disease entities, lesional status, or anatomic resection sites fail to address the overall outcome in an unselected group of patients making it difficult to understand the true impact or indications of epilepsy surgery.

#### 4.2.1. Resection Sites

In our study, the resection site was a powerful predictor of seizure recurrence, with extratemporal resections carrying nearly a 4.2-fold higher risk of seizure recurrence than temporal resections. These results are in keeping with prior studies, which showed seizure freedom in the range of 60–70% and 30–50% after temporal lobectomy and extratemporal resections, respectively [3, 18, 23]. Our study adds longitudinal follow-up data, demonstrating that the patients undergoing temporal resection experience significantly better outcome at each follow-up interval during the 5 years. At last follow-up, 91% (49 of 54) of patients who had temporal resections were seizure-free, whereas only 62% (6 of 16) of patients who had extratemporal resection were seizure-free. Less favorable outcome after extratemporal resections is probably related to the inherent difficulty in localizing and resecting the widespread epileptogenic zones in contrast to the temporal resections, which tend to result in a more complete removal of the epileptogenic zone. In addition, proximity to eloquent cortex (such as somatosensory, speech, and visual cortices) makes the resection of extratemporal foci more challenging, leading to incomplete removal despite invasive monitoring. We did not find any difference in outcome between temporal and extratemporal resections at the 5-year follow-up interval, which is most likely attributable to the relatively small sample size for extratemporal resections at that interval.

#### 4.2.2. EEG Findings

In our study, the ictal EEG findings concordant with the resection site predicted favorable outcome over the long term. At last follow-up, 92% of patients (48/52) with concordant EEG and 56% of patients (9/16 patients) with discordant EEG were seizure-free. This is along the lines of prior studies showing the value of EEG as a predictor of postoperative seizure outcome in the presence or absence of MRI abnormalities in patients with temporal or neocortical epilepsies [23, 25, 26].

In our study, interictal EEG was not a predictor of seizure recurrence, which is along the lines of the conclusions from a prior meta-analysis [23]. Studies are conflicting as to whether interictal EEG findings predict seizure recurrence [8, 11, 23]. This may be partly attributable to the variations in the definition of concordance between the interictal discharges and the resection site. Concordant interictal findings were defined as exclusive discharges in single brain regions, absence of generalized spikes, or occurrence of >70% lateralized discharges among various studies [8, 27, 28]. While it is intuitive to think that ictal and interictal EEG findings would be predictive of seizure outcome, our study showed that only the ictal EEG was a predictor of outcome. It is possible that ictal and interictal findings, taken together, might be predictive of outcome, but we did not specifically address that issue. Our results suggest the continued need for ictal recordings, rather than just the interictal data, for planning resection.

#### 4.2.3. MRI Findings

Our results are in agreement with other studies that have demonstrated that nonlesional MRI can be associated with an outcome as good as lesional MRI provided the scalp EEG findings are concordant with other functional studies and the planned resection site [9, 12, 29–31]. The relatively good outcome in patients with nonlesional MRI in our study is not surprising. Among 17 patients with nonlesional MRI, the postoperative pathology showed gliosis, nonspecific changes, and MTS in 11, 5, and 1 patients, respectively. Many of the patients with nonlesional MRI showed localizing findings on other studies, concordant with the intended surgical site; for example, 9/11 patients (82%) had PET abnormalities, 10/13 patients had (77%) SPECT abnormalities, and 13/18 patients (72%) had ictal EEG findings that were concordant. Thus, electrophysiological and non-MRI imaging studies strongly supported a well-localized epileptogenic zone in these patients. Our findings suggest that patients with nonlesional MRI can be good candidates for surgery as long as other data are concordant.

#### 4.2.4. Other Nonpredictors of Seizure Recurrence

Besides interictal EEG and MRI findings, the other nonpredictors of seizure recurrence in our study were age, gender, history of SGTC seizures, epilepsy duration, preoperative seizure frequency, number of AEDs, Wada memory lateralization, and lesion pathology. It is well established that gender is not predictive of outcome, but the literature is conflicting as to whether the other characteristics have predictive value [32, 33]. The discrepancies may be due to different patient populations, study methods, follow-up intervals, and classification methods.

Our study has a few limitations. Because of its retrospective nature, we were unable to determine if there were discrepancies in selecting patients for surgery. However, all the patients were discussed in a multidisciplinary conference, which ensured at least some degree of uniformity. The number of patients completing the long-term follow-up beyond 5 years was smaller due to loss to follow-up. We were unable to investigate the mechanisms of seizure recurrence because only a few patients with postoperative seizure recurrence underwent follow-up EEG or video-EEG evaluation. We were also unable to ascertain how many patients remained seizure- and aura-free because of inconsistencies in documentation. Prospective studies in larger cohorts are needed for a better understanding of the pathogenesis of seizure recurrence after epilepsy surgery. Nevertheless, our study demonstrates that epilepsy surgery is beneficial in intractable epilepsy and that temporal resection and concordant ictal EEG are the major determinants of favorable outcome over long-term follow-up.

## Figures and Tables

**Table 1:** Demographic and clinical characteristics of the cohort (70 patients).

Gender	Male 32 (46%); female 38 (54%)
Mean age, years (range)	39 (21–64)
Mean epilepsy duration, years (range)	18 (1–57)
History of secondary generalized tonic-clonic seizures, *n* (%)	46 (66%)
Mean number of antiepileptic drugs tried (range)	5 (1–10)
Mean follow-up, months (range)	48 (24–87)
Resection site, *n* (%)	Temporal, 54 (77%); extratemporal, 16 (23%)
Number of extraoperative invasive monitoring, *n* (%)	12 (17%)
Number of intraoperative electrocorticography, *n* (%)	41 (59%)
Ictal EEG, *n* (%)	Concordant, 52 (74%); discordant, 16 (23%); inconclusive, 2 (3%)
Interictal EEG, *n* (%)	Concordant, 37 (53%); discordant, 25 (36%); normal, 8 (11%)
MRI, *n* (%)	Abnormal 53 (76%); normal 17 (24%)
Number of patients who had Wada test, *n* (%)	52 (74%)
Wada memory lateralization, *n* (%)	Concordant 44 (85%); discordant 8 (15%)
Pathology, *n* (%)	Mesial temporal sclerosis or gliosis 45 (64%); benign tumor 7 (10%); vascular lesion 4 (4%); other 7 (10%); normal 7 (10%)

**Table 2:** Initial seizure recurrence pattern and subsequent outcome.

	Early recurrence (≤2 years)	Late recurrence (>2 years)	*p*
Total number of patients, *n*	18	4	<0.05
Number of patients with continued seizures at subsequent follow-up, *n*	15	1	
Number of patients seizure-free at subsequent follow-up, *n*	3	3	

**Table 3:** Predictors of seizure recurrence over 5 years of follow-up.

Predictor	Number of patients analyzed (*p* values)			
	2 years	3 years	4 years	5 years
Age (<30 versus ≥30 years)	70 (0.11)	50 (0.12)	36 (0.2)	27 (0.63)
Gender	70 (0.39)	50 (0.07)	36 (0.24)	27 (0.68)
History of GTC seizures	70 (0.08)	50 (0.73)	36 (0.2)	27 (0.05)
Epilepsy duration (<10 versus ≥10 y)	69 (0.13)	49 (0.07)	35 (1)	26 (1)
Seizure frequency (<10/m versus ≥10/m)	63 (0.5)	44 (0.47)	35 (0.51)	26 (0.63)
Number of AEDs (<5 versus ≥5)	68 (0.24)	49 (1)	35 (0.56)	26 (0.64)
MRI (normal versus abnormal)	70 (0.21)	50 (0.17)	36 (0.13)	27 (0.39)
Interictal EEG (concordant versus discordant)	62 (0.17)	43 (0.2)	31 (0.46)	25 (0.67)
Ictal EEG (concordant versus discordant with resection)	**68 (<0.01)**	**48 (0.01)**	**34 (0.02)**	**27 (0.01)**
Resection (temporal versus extratemporal)	**70 (0.04)**	**50 (0.02)**	**36 (0.04)**	27 (0.13)
Wada memory lateralization (contralateral versus other)	52 (0.27)	38 (1)	27 (0.17)	19 (0.42)
Pathology (MTS, gliosis, tumor, vascular, and other)	70 (0.6)	50 (0.38)	36 (0.09)	27 (0.42)

AED: antiepileptic drug; MTS: mesial temporal sclerosis; GTC: secondary generalized tonic-clonic seizures.

**Table 4:** Predictors of seizure recurrence: multivariate analysis.

	Risk ratio	95% CI	*p* value
Resection (temporal versus extratemporal)	4.2	1.5–11	<0.01
Ictal EEG (concordant versus discordant)	5.6	2.0–15.7	<0.01

CI: confidence interval.

## References

[1] Theodore W. H., Spencer S. S., Wiebe S. (2006). Epilepsy in North America: a report prepared under the auspices of the global campaign against epilepsy, the International Bureau for Epilepsy, the International League Against Epilepsy, and the World Health Organization. *Epilepsia*.

[2] Kwan P., Brodie M. J. (2000). Early identification of refractory epilepsy. *The New England Journal of Medicine*.

[3] Engel J., McDermott M. P., Wiebe S. (2012). Early surgical therapy for drug-resistant temporal lobe epilepsy: a randomized trial. *The Journal of the American Medical Association*.

[4] Wiebe S., Blume W. T., Girvin J. P., Eliasziw M. (2001). A randomized, controlled trial of surgery for temporal-lobe epilepsy. *The New England Journal of Medicine*.

[5] Spencer S. S., Berg A. T., Vickrey B. G. (2005). Predicting long-term seizure outcome after resective epilepsy surgery: the multicenter study. *Neurology*.

[6] Thom M., Mathern G. W., Cross J. H., Bertram E. H. (2010). Mesial temporal lobe epilepsy: how do we improve surgical outcome?. *Annals of Neurology*.

[7] Radhakrishnan K., So E. L., Silbert P. L. (2003). Prognostic implications of seizure recurrence in the first year after anterior temporal lobectomy. *Epilepsia*.

[8] Jeong S.-W., Lee S. K., Hong K.-S., Kim K.-K., Chung C.-K., Kim H. (2005). Prognostic factors for the surgery for mesial temporal lobe epilepsy: longitudinal analysis. *Epilepsia*.

[9] Sylaja P. N., Radhakrishnan K., Kesavadas C., Sarma P. S. (2004). Seizure outcome after anterior temporal lobectomy and its predictors in patients with apparent temporal lobe epilepsy and normal MRI. *Epilepsia*.

[10] Junna M. R., Buechler R., Cohen-Gadol A. A. (2013). Prognostic importance of risk factors for temporal lobe epilepsy in patients undergoing surgical treatment. *Mayo Clinic Proceedings*.

[11] Radhakrishnan K., So E. L., Silbert P. L. (1998). Predictors of outcome of anterior temporal lobectomy for intractable epilepsy: a multivariate study. *Neurology*.

[12] Lazow S. P., Thadani V. M., Gilbert K. L. (2012). Outcome of frontal lobe epilepsy surgery. *Epilepsia*.

[13] Elsharkawy A. E., Alabbasi A. H., Pannek H. (2009). Long-term outcome after temporal lobe epilepsy surgery in 434 consecutive adult patients. *Journal of Neurosurgery*.

[14] Foldvary N., Klem G., Hammel J., Bingaman W., Najm I., Lüders H. (2001). The localizing value of ictal EEG in focal epilepsy. *Neurology*.

[15] Risinger M. W., Engel J., Van Ness P. C., Henry T. R., Crandall P. H. (1989). Ictal localization of temporal lobe seizures with scalp/sphenoidal recordings. *Neurology*.

[16] Ebersole J. S., Pacia S. V. (1996). Localization of temporal lobe foci by ictal EEG patterns. *Epilepsia*.

[17] Engel J., Van Ness P., Rasmussen T. B., Ojemann L. M., Engel J. (1993). Outcome with respect to epileptic seizures. *Surgical Treatment of the Epilepsies*.

[18] Téllez-Zenteno J. F., Dhar R., Wiebe S. (2005). Long-term seizure outcomes following epilepsy surgery: a systematic review and meta-analysis. *Brain*.

[19] Jeha L. E., Najm I. M., Bingaman W. E. (2006). Predictors of outcome after temporal lobectomy for the treatment of intractable epilepsy. *Neurology*.

[20] Yoon H. H., Kwon H. L., Mattson R. H., Spencer D. D., Spencer S. S. (2003). Long-term seizure outcome in patients initially seizure-free after resective epilepsy surgery. *Neurology*.

[21] Najm I., Jehi L., Palmini A., Gonzalez-Martinez J., Paglioli E., Bingaman W. (2013). Temporal patterns and mechanisms of epilepsy surgery failure. *Epilepsia*.

[22] Goellner E., Bianchin M. M., Burneo J. G., Parrent A. G., Steven D. A. (2013). Timing of early and late seizure recurrence after temporal lobe epilepsy surgery. *Epilepsia*.

[23] Tonini C., Beghi E., Berg A. T. (2004). Predictors of epilepsy surgery outcome: a meta-analysis. *Epilepsy Research*.

[24] McIntosh A. M., Wilson S. J., Berkovic S. F. (2001). Seizure outcome after temporal lobectomy: current research practice and findings. *Epilepsia*.

[25] Tatum W. O., Benbadis S. R., Hussain A. (2008). Ictal EEG remains the prominent predictor of seizure-free outcome after temporal lobectomy in epileptic patients with normal brain MRI. *Seizure*.

[26] Yun C.-H., Lee S. K., Lee S. Y., Kim K. K., Jeong S. W., Chung C.-K. (2006). Prognostic factors in neocortical epilepsy surgery: multivariate analysis. *Epilepsia*.

[27] Jeha L. E., Najm I., Bingaman W., Dinner D., Widdess-Walsh P., Lüders H. (2007). Surgical outcome and prognostic factors of frontal lobe epilepsy surgery. *Brain*.

[28] Noe K., Sulc V., Wong-Kisiel L. (2013). Long-term outcomes after nonlesional extratemporal lobe epilepsy surgery. *JAMA Neurology*.

[29] Bell M. L., Rao S., So E. L. (2009). Epilepsy surgery outcomes in temporal lobe epilepsy with a normal MRI. *Epilepsia*.

[30] Holmes M. D., Born D. E., Kutsy R. L., Wilensky A. J., Ojemann G. A., Ojemann L. M. (2000). Outcome after surgery in patients with refractory temporal lobe epilepsy and normal MRI. *Seizure*.

[31] LoPinto-Khoury C., Sperling M. R., Skidmore C. (2012). Surgical outcome in PET-positive, MRI-negative patients with temporal lobe epilepsy. *Epilepsia*.

[32] Zaatreh M. M., Spencer D. D., Thompson J. L. (2002). Frontal lobe tumoral epilepsy: clinical, neurophysiologic features and predictors of surgical outcome. *Epilepsia*.

[33] Zaatreh M. M., Firlik K. S., Spencer D. D., Spencer S. S. (2003). Temporal lobe tumoral epilepsy: characteristics and predictors of surgical outcome. *Neurology*.

